# Mechanical properties of carbon nanotube/polymer composites

**DOI:** 10.1038/srep06479

**Published:** 2014-10-01

**Authors:** B. Arash, Q. Wang, V. K. Varadan

**Affiliations:** 1 Department of Mechanical Engineering, University of Manitoba, Winnipeg, MB, R3T 5V6, Canada; 2 Department of Electrical Engineering, University of Arkansas, Fayetteville, AR, 72701-1201, U.S

## Abstract

The remarkable mechanical properties of carbon nanotubes, such as high elastic modulus and tensile strength, make them the most ideal and promising reinforcements in substantially enhancing the mechanical properties of resulting polymer/carbon nanotube composites. It is acknowledged that the mechanical properties of the composites are significantly influenced by interfacial interactions between nanotubes and polymer matrices. The current challenge of the application of nanotubes in the composites is hence to determine the mechanical properties of the interfacial region, which is critical for improving and manufacturing the nanocomposites. In this work, a new method for evaluating the elastic properties of the interfacial region is developed by examining the fracture behavior of carbon nanotube reinforced poly (methyl methacrylate) (PMMA) matrix composites under tension using molecular dynamics simulations. The effects of the aspect ratio of carbon nanotube reinforcements on the elastic properties, i.e. Young's modulus and yield strength, of the interfacial region and the nanotube/polymer composites are investigated. The feasibility of a three-phase micromechanical model in predicting the elastic properties of the nanocomposites is also developed based on the understanding of the interfacial region.

The outstanding electrical, mechanical, and thermal properties of carbon nanotubes (CNTs)^1^,^2^ have made them among the most promising materials in a wide range of applications such as nano-sensors and atomic transportation^3^,^4^. In addition, the excellent mechanical properties of CNTs, such as ultra-high Young's modulus around 1 TPa^5^ and tensile strengths varying from 11–63 GPa^6^, are promising ultra-high-strength reinforcements in high-performance polymer matrix composites.

In order to facilitate the development of reinforced polymer composites and the design of the materials, the bulk mechanical properties of the materials must be determined. Although some experiments have been conducted on material properties of CNT reinforced composites^7^,^8^,^9^, scattered data on the nano-reinforced composites have been reported in literature^10^,^11^,^12^. The main reasons for the inconsistency are attributed to drawbacks in the uniform alignment of CNT reinforcements and forming proper interfacial bonding between matrix and CNTs during the mixing process of the composites. Molecular dynamics (MD) simulations, however, can provide alternative methods to generate detailed information, such as stress-strain behavior and the interfacial interactions between matrix and CNTs. Hence, MD is indispensable in understanding mechanical properties of CNT/polymer composites^13^,^14^,^15^. In addition, MD simulation studies may enable interpretations of experimental results and even a route to new designs of laboratory tests. Frankland et al.^16^ investigated stress–strain curves of single-walled CNTs (SWCNTs) reinforced polyethylene matrix composites. They reported that long SWCNTs enhance the stiffness of nanocomposites obviously; however, no significant enhancement was observed for short SWCNTs. Zhu et al.^17^ utilized MD simulations to study the stress–strain behavior of single-walled CNTs (SWCNTs) reinforced epoxy Epon 862 composites. Their simulation results showed that a (10, 10) SWCNT with an aspect ratio of around 2.15 embedded along the longitudinal direction of an epoxy Epon 862 matrix with a size of 4.028 × 4.028 × 6.109 nm^3^ can increase the Young's modulus of Epon 862 composites as high as 20%. Han and Elliott^18^ used MD simulations to model CNT reinforced composites made of a (10, 10) SWCNT with an effectively infinite length embedded in two different amorphous polymer matrices of poly (methyl methacrylate) (PMMA) and poly{(m-phenylenevinylene)-co-[(2,5-dioctoxy-p-phenylene) vinylene]} (PmPV). Based on their simulation results, the Young's moduli in the longitudinal direction of CNT/PMMA and CNT/PmPV composites with a CNT volume fraction of 17% are 138.9 GPa and 145.6 GPa, respectively. Molecular simulations conducted by Mokashi et al.^19^ on mechanical properties of long SWCNT reinforced amorphous polyethylene composites reveal that the Young's modulus of the composites is 82 GPa at an 11.25% CNT volume fraction which is about 25 times of that for pure amorphous polyethylene. Molecular simulation studies on mechanical properties of CNT reinforced Poly (vinylidene fluoride) (PVDF) matrix composites^20^ showed that an introduction of (5, 5) SWCNTs with a length of 2 nm can increase the Young's modulus of the systems by 1 GPa.

In the abovementioned molecular studies, the effect of CNT reinforcements on mechanical properties of polymer composites has been investigated. However, mechanical properties reported in the studies are scattered and may not be directly used in a continuum-based framework for design purposes as some effects are still obscure such as the geometries of CNTs. The major reason for the inapplicability of the results in a continuum framework is because the interfacial separation and sliding between reinforcements and matrices owing to the weakened bonding formed between CNTs and polymer matrices have not been accounted systematically. As a result, the contribution of the interfacial region on the overall mechanical properties of CNT/polymer composites cannot be addressed in the framework for future designs.

In view of the above problem, analytical approaches based on continuum micromechanics models in conjunction with molecular simulations have been developed to study the contribution of the interphase zone in the overall elastic properties of nanocomposites^21^,^22^,^23^,^24^,^25^,^26^. Based on the pullout simulations^21^, the interfacial shear strength between (10, 10) CNTs and an epoxy resin matrix was calculated to be up to 75 MPa, indicating that there is an effective stress transfer from the epoxy resin to nanotubes. Liao and Li^22^ studied the interfacial characteristics of CNT-reinforced polystyrene composites using a CNT pullout simulation. They reported that the interfacial shear stress of between CNT reinforcements with an outer diameter of 1.33 nm and a polystyrene matrix is about 160 MPa that is significantly higher than most carbon fiber reinforced polymer composites. In another study^23^, CNT pull-out from the polymer matrix was modeled with MD simulations to investigate the effects of the matrix density, chemical cross-links in the interface and geometrical defect in CNTs on the interfacial shear strength of CNT reinforced polyethylene composites. Although the simulation studies on the pullout of CNT reinforcements from polymer matrices evidences the load transfer capability from polymer matrices to nanotubes, they do not provide mechanical properties of the nanocomposites and the interfacial region such Young's modulus and yield stress. In this regard, other models were developed to measure the properties. Tsai et al.^24^ calculated the non-bonded gap and the non-bonded energy between the CNTs and the surrounding polyimide polymer. They developed a three-phase micromechanical model comprising the CNTs, effective interphase, and polyimide polymer. Yang et al.^25^ developed a multiscale model to consider the effect of CNT sizes and weakened bonding at the interface on the effective elastic stiffness of CNT/polymer nanocomposites using MD simulations and continuum micromechanics. In the study, the constitutive relations were modified to study the interfacial separation by adopting a linear spring layer between the filler and matrix. Wang^26^ developed a continuum modeling of the van der Waals (vdW) interactions between CNTs and polymers as mechanical spring elements by fitting the molecular mechanics results. Based on the model, the interaction can be modeled as an external pressure on the tube walls. It is noted that the constitutive relations modified in the proposed models for the interfacial region are limited to a linear spring layer between reinforcements and matrix only in the radial direction. Because of the limitation of the models, the effect of bonding, debonding and relative sliding between fibers and matrix on the mechanical properties cannot be modeled properly. On the other hand, since the interfacial shear stress between CNT reinforcements and matrix through the vdW interactions along the nanotubes axial direction was ignored in the proposed models, the prediction of mechanical properties of the CNT-composites cannot be reliable and properly identified. Furthermore, changes in the adhesion behaviors at large strains caused by nonlinear effects between CNT fibers and surrounding polymer matrix in both radial and longitudinal directions were also neglected in the models. Thus, a more comprehensive molecular level understanding of the reinforcing mechanism in predicting overall elastic properties of the interfacial region with a thorough consideration of nanoscale effects are necessary to fulfill design, synthesis, and characterization of CNT/polymer nanocomposites.

In this work, a new method for evaluating the elastic properties of the interfacial region between CNT reinforcements and polymer matrices in composites is developed via molecular studies on the mechanical behavior of CNT/PMMA composites subjected to tension. The effect of the aspect ratio of CNT fibers on the elastic properties of the interfacial region and the overall stiffness of the nanocomposites is investigated in details. The feasibility of a developed three-phase micromechanical model in prediction of elastic properties of the nanocomposites based on the understanding of the interfacial region is examined through a verification process with molecular simulation results.

## Results

### Calculation of the elastic moduli

**Figure 1:**
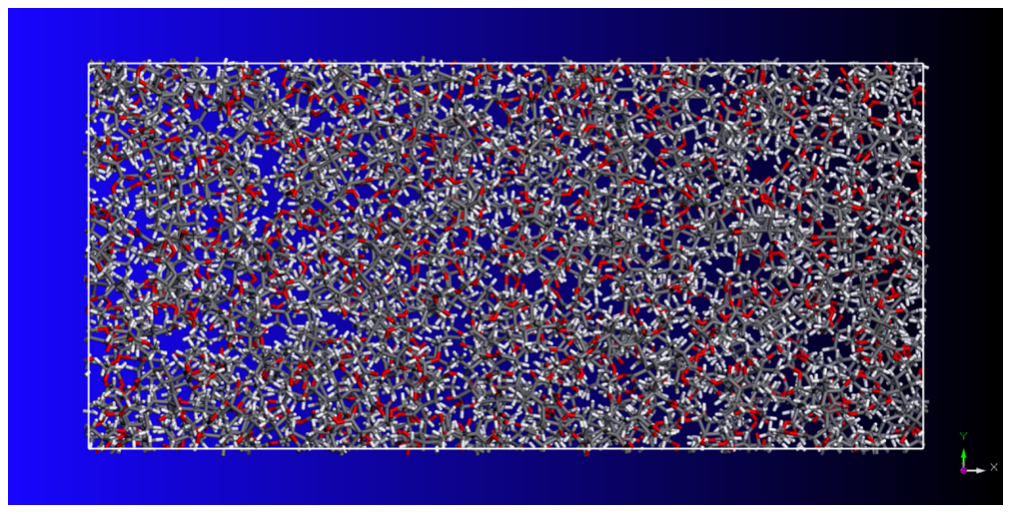
Molecular unit cell model of PMMA matrix with a size of 8.0 × 3.7 × 3.7 nm^3^ and a mass density of 1.17 *g*/*cm*^3^.

In order to investigate mechanical properties of CNT/PMMA composites, the Young's modulus of PMMA polymer ((CH_2_ = C[CH_3_]CO_2_CH_3_)_n_) is first calculated. A simulation unit cell with a size of 8.0 × 3.7 × 3.7 nm^3^ and periodic boundary conditions that contains amorphous PMMA polymer with a mass density of 1.17 *g/cm*^3^ is initially constructed. The PMMA polymer is generated by 10 repeated monomer units. The constant-strain energy minimization method is applied to calculate the elastic modulus of the polymer system. After an initial energy minimization, a small strain of 0.05% is applied to the periodic structure in the longitudinal direction (*x*-direction) as illustrated in Figure 1. The application of the tensile strain is accomplished by uniformly expanding the dimensions of the simulation cell in the direction of the deformation and re-scaling the new coordinates of the atoms to fit within the new dimensions. After each increment of the applied strain, the potential energy of the structure is re-minimized keeping the lattice parameters fixed. The total potential energy and the interaction energy are then measured in the minimized structure. This process is repeated for a series of strains. Finally, the variation of the measured potential energies versus applied strain is used to calculate the effective Young's moduli of the interfacial region and composite as 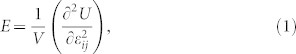
where U is the potential energy, *V* is the unit volume and *ε* is strain. The Young's modulus of the amorphous PMMA matrix from the present simulations is obtained to be 2.86 GPa, while the available experimental results vary from 2.24 to 3.8 GPa^18^.

Next, to investigate the mechanical properties of CNT/PMMA composites, we propose a method for measuring the mechanical properties of the interfacial region between CNTs and surrounding matrix material. The principle of the proposed method is based on a calculation of the interaction energy from the energy difference between the total internal energy of the composite and the sum of the energies of individual molecules: 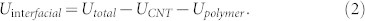
where *U_total_* is the total potential energy of the composite, *U_CNT_* is the potential energy of CNT reinforcements, and *U_polymer_* is the potential energy of polymer matrix. It is noteworthy that in the absence of covalent chemical bonding, the interfacial bond strength comes from the electrostatic and vdW forces in the molecular system. Then, the effective Young's moduli of the composite and the interfacial region are respectively calculated from the second derivative of the total potential energy (*U_total_*) and the interaction potential energy (*U_interfacial_*) with respect to the tensile strain (*ε*) by using Eq. (1). In the following simulations, the Young's moduli of CNT/PMMA composites and the interfacial region between CNTs and matrix are respectively denoted by *E_c_* and *E_i_*, and the yield stress of the composite and the interfacial region are respectively denoted by *σ_yc_* and *σ_yi_*.

**Figure 2:**
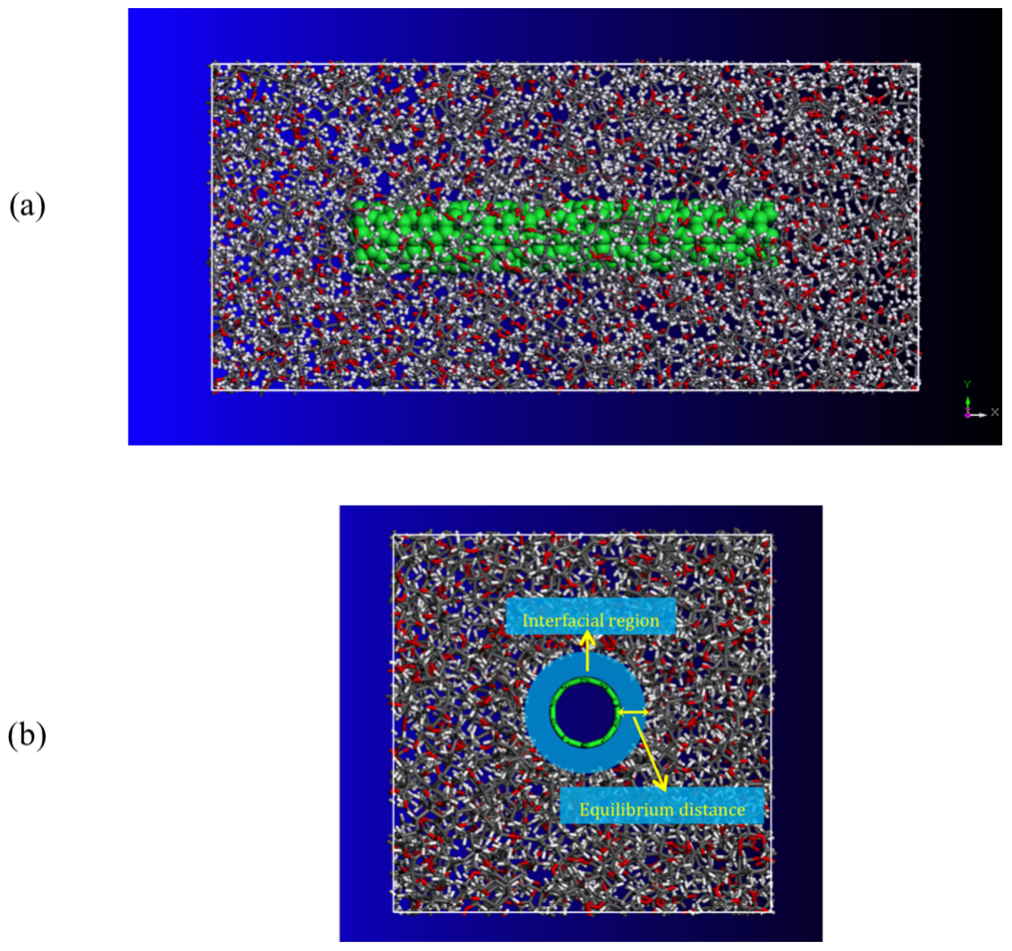
Molecular unit cell model of PMMA matrix with a size of 8.0 × 3.7 × 3.7 nm^3^ reinforced with a (5, 5) CNT with a length of 5 nm. The mass density of the polymer matrix is 1.17 *g*/*cm*^3^. (a) side view, and (b) cross section view.

**Figure 3:**
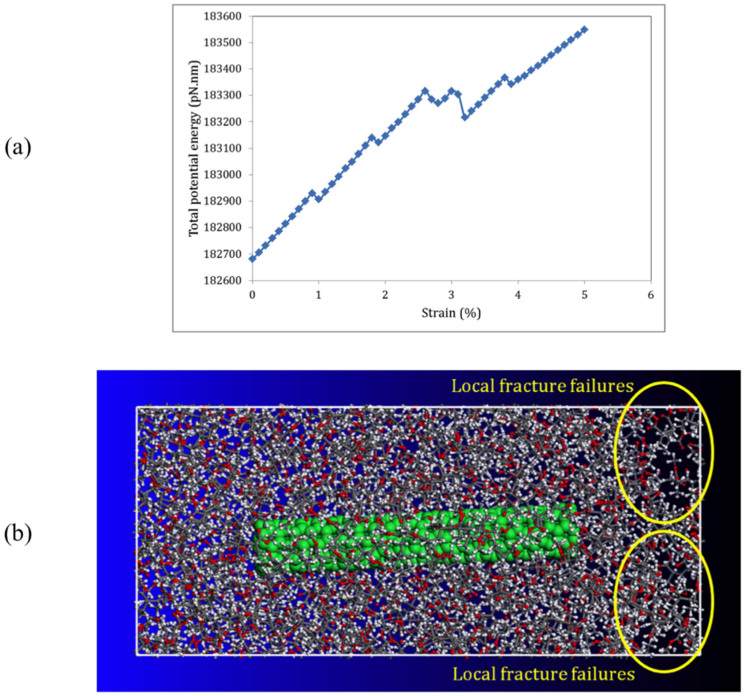
The mechanical behavior of the CNT/PMMA composite under tensile strain in the longitudinal direction. (a) the variation of the total potential energy of the composite versus strain, (b) a snapshots of the composite at strain of 1% demonstrating local fracture failures in the composite due to the tensile loading.

In order to examine the effectiveness and applicability of the method, we conduct the same process of applying tensile strain in the x-direction to a unit cell of amorphous PMMA polymer with a size of 8.0 × 3.7 × 3.7 nm^3^ and periodic boundary conditions reinforced by a (5, 5) CNT with a length of 5 nm as illustrated in Figure 2(a). In the study, the equilibrium distance between CNTs and polymer matrix, which varies from 0.29 to 0.31 nm, owing to the vdW interactions between them is taken as the interfacial region as illustrated in Figure 2(b). The mass density of the PMMA polymer matrix is set to 1.17 *g/cm*^3^. The variation of the total potential energy of the CNT/PMMA composite under axial tensile strains is presented in Figure 3(a) from which the yield strain is obtained to be *ε_Y_* = 0.9%. A Snapshot of the failure in the (5, 5) CNT/PMMA composite at the tensile strain of 1% is illustrated in Figure 3(b), from which a local fracture failure is observed in the composite due to the tensile loading.

**Figure 4:**
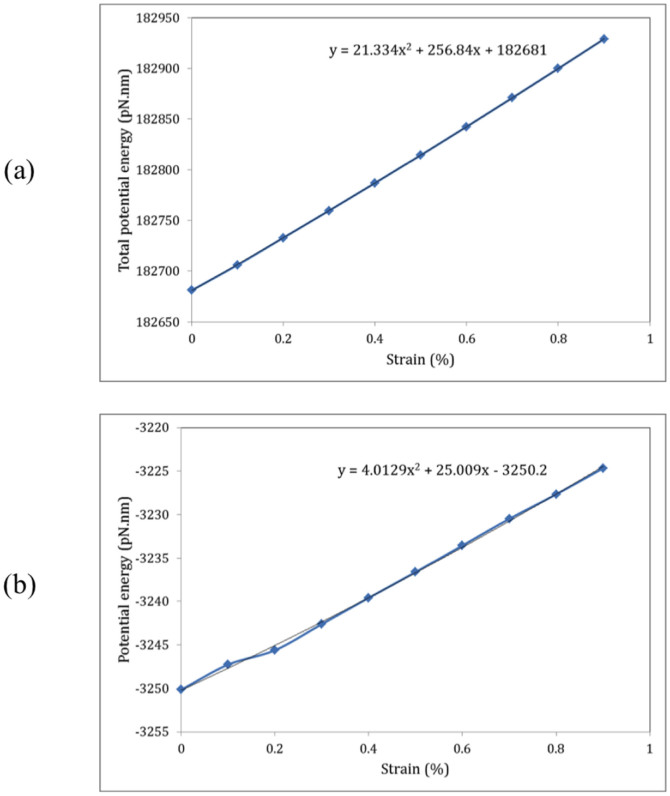
The mechanical behavior of the CNT/PMMA composite under tensile strain in the longitudinal direction. (a) the variation of the total potential energy of the composite versus strain, (b) the variation of the interaction potential energy of the interfacial region between the (5, 5) CNT reinforcement and PMMA polymer matrix.

To further investigate mechanical properties of the CNT/PMMA composite, we respectively zoom in the variation of total potential energy and the interaction energy between the CNT and polymer matrix in Figures 4(a) and 4(b) from *ε* = 0 to *ε* = 0.9%. From Figure 4(a), the Young's modulus of the PMMA polymer composite reinforced by the (5, 5) CNT can be calculated to be *E_c_* = 3.90 *GPa* as presented in Table 1, revealing a percentage increase of 36% in the stiffness of the PMMA polymer owing to the application of the (5, 5) CNT reinforcement. Similarly, the Young's modulus of the interfacial region is obtained to be *E_i_* = 0.73 *GPa* from Figure 4(b). The yield stress can also be obtained from *σ_Y_* = *Eε_Y_* by which the yield stress of the CNT/PMMA composite and the interfacial region are respectively calculated to be *σ_Yc_* = 35.06 *MPa* and *σ_Yi_* = 6.60 *MPa* as presented in Table 1. The simulation results indicate a slight increase (less than 3%) in Young's modulus and the yield stress of the PMMA/CNT composite with an increase in the length of polymer chains from 10 to 30 repeated monomer units. For example, Young's modulus of the nanocomposite increases from 3.90 to 4.01 GPa with an increase in the length of polymer chains from 10 to 30 repeated monomer units.

Hence, the proposed method enables a direct derivation of the mechanical properties, i.e. the Young's modulus, the yield stress and the yield strain, of the interfacial region from molecular simulations. The method also allows the consideration of the influence of nanoscale effects on the mechanical properties since all parameters affecting the amount of the interfacial energy, such as relative sliding between CNT reinforcements and matrix and the transverse deformation of nanotubes, are included in the variation of the interaction potential energy.

### Effect of the aspect ratio of CNTs on mechanical properties

**Figure 5:**
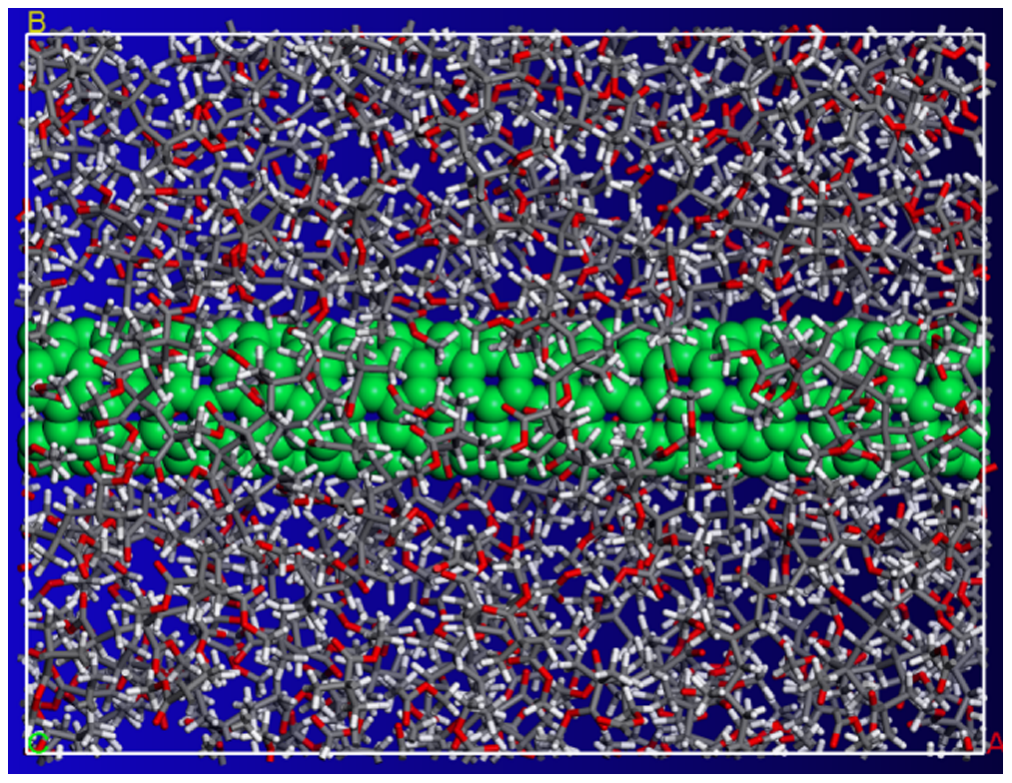
Molecular unit cell model of PMMA matrix with a size of 5.0 × 3.7 × 3.7 nm^3^ reinforced with a (5, 5) CNT with a length of infinity. The mass density of the polymer matrix is 1.17 *g*/*cm*^3^.

To further explore the mechanical behavior of CNT/PMMA composites, the effect of the aspect ratio of the CNT reinforcements is presented in Table 2. The diameter of the (5, 5) CNT reinforcements is 0.68 nm and their length-to-diameter ratio (*L*/*d*) varies from 5 to ∞. The mass density of PMMA polymer matrix is set to be 1.17 *g*/*cm*^3^. In simulations of nanocomposite unit cells reinforced by CNTs with an infinite length, periodic boundary conditions are imposed in all directions and no end effect of the CNTs (such as a cap or hydrogenation) is considered, as depicted in Figure 5. Thus, the embedded CNT has infinite length.

From Table 2, the Young's modulus of PMMA polymer matrix reinforced by (5, 5) CNTs increases from 3.9 GPa to 4.73 GPa and 6.85 GPa with an increase in the aspect ratio of the CNTs from 7.23 to 14.71 and 22.05. The simulation results show a percentage increase of 21% and 75% in the stiffness of the CNT/PMMA composite with an increase in the aspect ratio of the CNTs from 7.23 to 14.71 and 22.05. In addition, the Young's modulus of the nanocomposite with an infinite long CNT reinforcement significantly increases to 46.73 GPa, which is respectively 12 and 16 times stiffer than a CNT/PMMA composite with a fiber with the aspect ratio of 7.23 and a pure PMMA polymer material. The measured Young's modulus for PMMA matrix composite reinforced by an infinite long CNT is in excellent agreement with a value of 45.94 GPa reported in Ref. 18, where the fracture volume of nanotube is 5.51%.

While the Young's modulus of the nanocomposite significantly enhances by increasing the length-to-diameter ratio of CNTs, the Young's modulus of the interfacial region relatively experiences a slight increase from 0.73 GPa to 0.95 GPa and 1.10 GPa with an increase in the aspect ratio of the CNT fibers from 5 to 10 and 15. The Young's modulus of the interfacial region with an infinite long CNT reinforcement increases to 2.34 GPa, showing an increase of 220% in the stiffness of the interfacial region compared to PMMA polymer matrix reinforced by a CNT with the aspect ratio of 7.23. The strengthening of CNT/polymer interfacial bonding with an increase in the aspect ratio of CNT fibers results in the increase of stress transfer between CNTs and polymer, which in turn, leads to higher composite stiffness and strength as observed in molecular simulations.

### Micromechanics models

**Figure 6:**
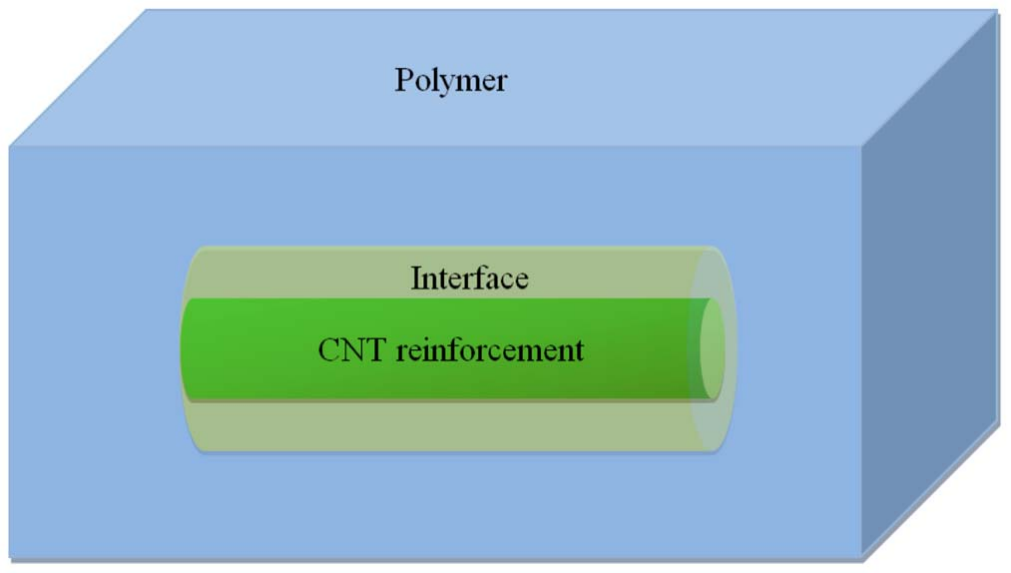
Schematic illustration of effective interface micromechanics model.

The mechanical behaviors observed in molecular simulations can be interpreted by developed micromechanical continuum models. Micromechanical models provide simple approaches to predict overall properties of composites using geometries and properties of individual phases that constitute the materials. Two-phase micromechanical models, such as the Mori–Tanaka method^27^,^28^, assume that only matrix and reinforcement phases exist in a composite material and they are perfectly bonded to each other. Although the models are precise enough when the size of reinforcements is in the order of micrometers or higher, the reinforcement and adjacent polymer region were not accurately described^29^. Hence, they are inaccurate in predicting properties of the composites with shorter reinforcements. Due to the aforementioned drawbacks, the effective interface model that considers three phases co-existence (i.e., matrix, interfacial region and reinforcement) was developed^30^. The effective interface model was used to predict the elastic properties of composites with effective reinforcements with an interface of the same shape as the effective fibers as illustrated in Figure 6. The effective interface has a finite size surrounding the reinforcement, which is referred to as an interphase or an interfacial region. Based on this model, the bulk elastic stiffness of composite (*E_c_*) is predicted as 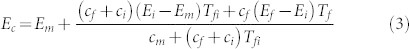
where *E_m_*, *E_i_* and *E_f_* are respectively the Young's moduli of matrix, interfacial region and fiber; and *c_m_*, *c_i_* and *c_f_* are the volume fraction of matrix, interfacial region and fiber. *T_f_* and *T_fi_* are the dilute strain concentration tensors given by^30^

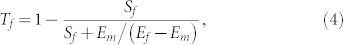
where *v* is Poisson's ratio of the matrix, *α* is the aspect ratio of the fiber (*l*/*d*) and 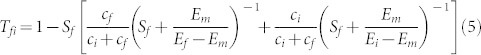
In Eq. (4), the Eshelby tensor, *S_f_*, is a function of the fiber aspect ratio and the matrix elastic constants given by^31^

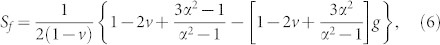
and 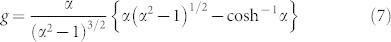
The key result of Eshelby is to show that within a fiber the strain is uniform. We now apply the three-phase theory in investigation of mechanical properties of CNT-PMMA composites based on the derivation of the interfacial region developed in the previous section. In order to use the micromechanical model, the Young's moduli of the interfacial region and PMMA polymer matrix are taken from molecular simulation results presented in Tables 1 and 2. The Young's modulus of a (5, 5) CNT reinforcement is also measured to be 1.65 TPa using MD simulations. It is noteworthy that in calculation of the Young's modulus of the CNT, the nanotube is supposed to be a solid bar with a cross sectional area of 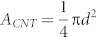. The process of applying tensile strain is the same as that described in the measurement of stiffness of CNT/PMMA composites.

**Figure 7:**
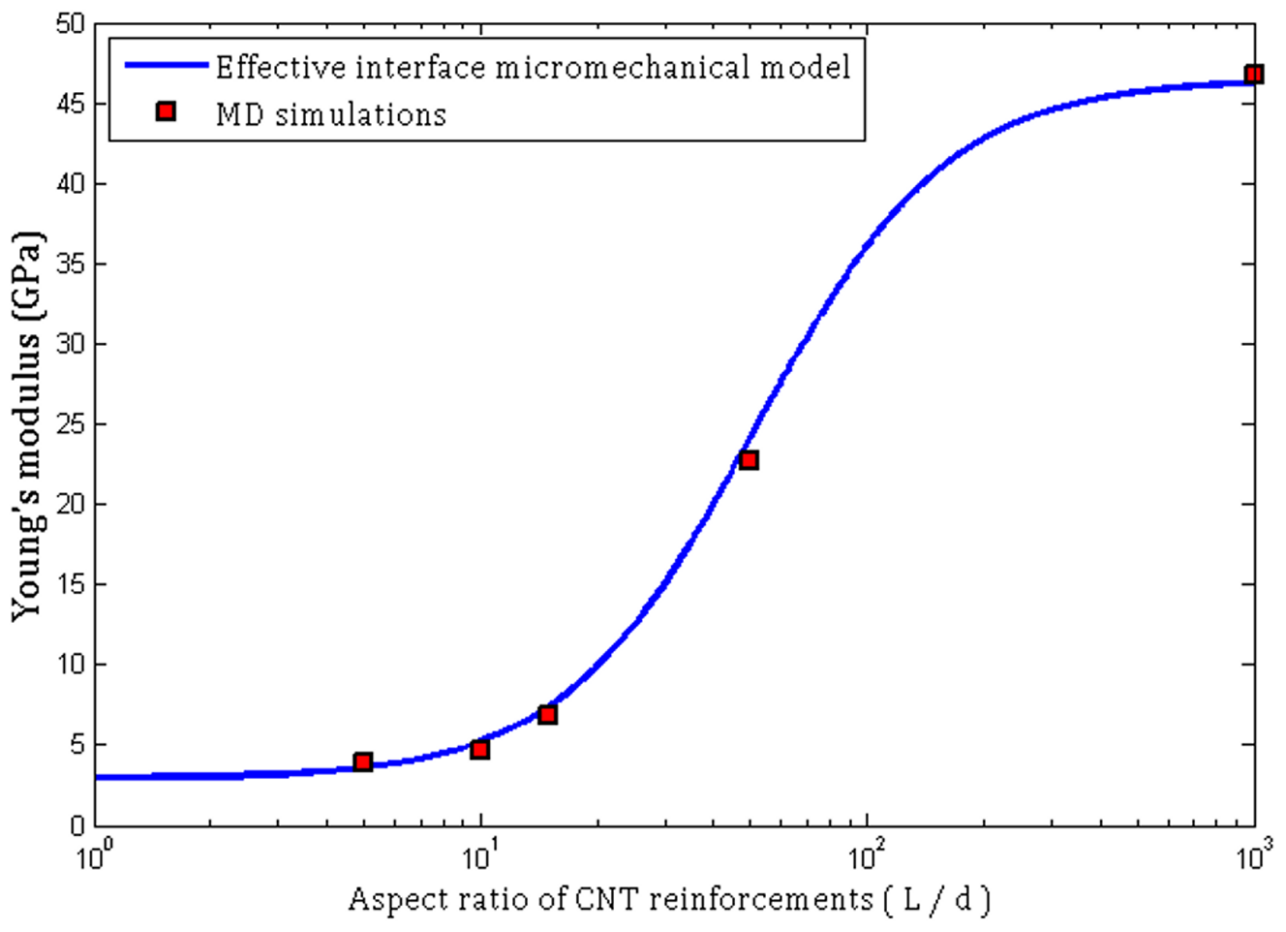
Micromechanical model predictions and molecular simulation results for the Young's modulus of (5, 5) CNT/PMMA composite materials.

Based on the above description, the Young's moduli of (5, 5) CNT/PMMA composites versus the aspect ratio of nanotube fibers (*L*/*d*) predicted by the effective interface model in Eq. (3) are compared to those obtained from MD simulations in Figure 7. From Figure 7, an excellent agreement between results obtained from the effective interface model and MD simulations is obtained. The Young's modulus of a PMMA composite unit cell reinforced by a (5, 5) CNT with an aspect ratio of 7.23 are respectively obtained to be 3.90 GPa and 3.57 GPa using MD simulations and the effective interface model, revealing a percentage difference of 9.2%. The percentage difference decreases to 7.8% at *L*/*d* = 22.05, where the Young's modulus of the CNT/PMMA composite predicted by molecular simulations and the effective interface mode are respectively 6.85 GPa and 7.39 GPa. For infinite long CNT fiber, the stiffness of the composite is respectively obtained to be 46.73 GPa and 46.56 GPa, indicating a percentage difference of 0.37%. It is concluded that the three-phase micromechanical model, incorporating the effect of interfacial region, could efficiently predict the stiffness of nanotube/polymer composites. Hence, it is expected that the effective interface model is applicable to nanometer-sized reinforcements. It is noteworthy that the key point in a successful application of the effective interface model is to determine mechanical properties of the interfacial region using molecular simulations.

## Discussion

The mechanical behavior of CNT/PMMA composite materials subjected to tensile loading is studied using MD simulations. A new method for the measurement of the elastic properties of the interfacial region between the CNT reinforcements and the PMMA polymer matrix is proposed. The effect of the aspect ratio of CNT fibers on the elastic properties of the nanocomposites and the interfacial region between nanotubes and the polymer matrix is explored. Simulation results demonstrate that the Young's modulus of a PMMA polymer matrix composite reinforced by an infinite long (5, 5) CNT significantly increases to 46.73 GPa, which is 16 times stiffer than a pure PMMA polymer material. In addition, the strength of CNT/polymer interfacial bonding increases with an increase in the aspect ratio of CNT fibers, which in turn, leads to a high composite stiffness. An effective continuum interface model based on the results from the interfacial region was developed and its feasibility in prediction of elastic properties of the nanocomposites is justified through a verification process with molecular simulation results.

## Methods

First, a two two-dimensional model of PMMA polymer with 10 repeated monomer units is constructed. An energy minimization is performed by using the conjugate-gradient method^32^ on the molecule to achieve a reasonable three-dimensional model. Partial charges of atoms were assigned using Qeq method^33^. Next, a number of the PMMA molecules are packed into a cubic lattice corresponding to a predefined density of 1.17 g/cm^3^ as illustrated in Figures 1 and 2. The generation is fulfilled by the Amorphous Cell Packing task in Accelrys Materials Studio 6.0. The module builds molecules in a cell with a Monte Carlo fashion, by minimizing close contacts between atoms, whilst ensuring a realistic distribution of torsion angles for a given force-field. In this work, we choose COMPASS force-field^34^, which is the first ab initio force-field that enables accurate and simultaneous prediction of a broad range of molecules and polymers. In the nonbonding terms, van der Waals interaction energy and coulombic interaction energy terms are included for the force-field. A potential cutoff of 1.5 nm is used in calculation of nonbonded interactions.

In order to find a global minimum energy configuration, we utilize the approach by Li and Mattice^35^ in the refinement procedure. Molecular simulations are initialized with a geometry optimization process to minimize the total energy of the system (i.e., a CNT surrounded polymer matrix as illustrated in Figure 2). Once the minimization process is completed, the system is then allowed to equilibrate over the constant volume and constant temperature (NVT) ensemble at the room temperature of 298 K for 50 ps. The simulation time step is set to be 1 fs in the NVT simulation, and the Andersen feedback thermostat^36^ is used for the system temperature conversion. An energy minimization with the convergence criteria of 0.00001 kcal/mol is then followed. After that, the temperature of the system is increased to 800 K and a further 100 ps of NVT dynamics is applied. It is followed by a further energy minimization. After the preparation of a CNT/PMMA composite material, the constant-strain minimization method is applied to the equilibrated system to measure the material properties of the composite. In simulations, an automatic recalculation of bonds is used to monitor bonding configurations and capture polymer bond breakage. The criteria used to calculate bonds are based on the distance between atoms and the element type. The calculate bonds tool creates bonds between two atoms if the sum of the covalent radii of the two atoms fulfills the bond-length criterion: TF_lower_ × ideal distance < distance < TF_upper_ × ideal distance. TF_lower_ is the lower tolerance factor; TF_upper_ is the upper tolerance factor; distance is the distance between the two atoms forming the bond. TF_lower_ and TF_upper_ are set to be 0.6 and 1.35, respectively. The ideal distance is also defined as the sum of the covalent radii of the two atoms. During simulations, the calculate bonds tool monitors bonds, and automatically recalculates bonds if the position of atoms changes. The MD simulations have been performed with the Forcite module of Materials Studio software package.

## Author Contributions

B.A. conducted the molecular simulations and theoretical analyses. Q.W. supervised the whole work and V.K.V. contributed to the manuscript preparation and discussions. These authors read and corrected the manuscript before the submission.

## Figures and Tables

**Table 1:** Mechanical properties of PMMA polymer, PMMA polymer matrix reinforced by a (5, 5) CNT with a length of 5 nm and the interfacial region

	PMMA polymer	(5, 5) CNT/PMMA	Interfacial region
Young's modulus (GPa)	2.86	3.90	0.73
Yield stress (MPa)	-	35.06	6.60
Yield strain (%)	-	0.9	0.9

**Table 2:** Mechanical properties of PMMA polymer, PMMA polymer matrix reinforced by a (5, 5) CNT with a length of 5 nm and the interfacial region

Aspect ratio of CNT (L/d)	CNT/PMMA	Interfacial region
7.23	3.90	0.73
14.71	4.73	0.95
22.01	6.85	1.10
∞	46.73 (45.94 [18])	2.34
